# Spatiotemporal regulation of the cough motor pattern

**DOI:** 10.1186/1745-9974-5-12

**Published:** 2009-12-22

**Authors:** Cheng Wang, Sourish Saha, Melanie J Rose, Paul W Davenport, Donald C Bolser

**Affiliations:** 1 Department of Physiological Sciences, College of Veterinary Medicine, University of Florida, Gainesville, Florida, 32610, USA; 2 Department of Statistics, College of Liberal Arts and Sciences, University of Florida, Gainesville, Florida, 32611, USA

## Abstract

The purpose of this study was to identify the spatiotemporal determinants of the cough motor pattern. We speculated that the spatial and temporal characteristics of the cough motor pattern would be regulated separately. Electromyograms (EMG) of abdominal muscles (ABD, rectus abdominis or transversus abdominis), and parasternal muscles (PS) were recorded in anesthetized cats. Repetitive coughing was produced by mechanical stimulation of the lumen of the intrathoracic trachea. Cough inspiratory (CT_I_) and expiratory (CT_E_) durations were obtained from the PS EMG. The ABD EMG burst was confined to the early part of CT_E _and was followed by a quiescent period of varying duration. As such, CT_E _was divided into two segments with CT_E1 _defined as the duration of the ABD EMG burst and CT_E2 _defined as the period of little or no EMG activity in the ABD EMG. Total cough cycle duration (CT_TOT_) was strongly correlated with CT_E2 _(r^2^>0.8), weakly correlated with CT_I _(r^2^<0.3), and not correlated with CT_E1 _(r^2^<0.2). There was no significant relationship between CT_I _and CT_E1 _or CT_E2_. The magnitudes of inspiratory and expiratory motor drive during cough were only weakly correlated with each other (r^2^<0.36) and were not correlated with the duration of any phase of cough. The results support: a) separate regulation of CT_I _and CT_E_, b) two distinct subphases of CT_E _(CT_E1 _and CT_E2_), c) the duration of CT_E2 _is a primary determinant of CT_TOT_, and d) separate regulation of the magnitude and temporal features of the cough motor pattern.

## Background

Cough is an important airway defensive behavior. It is characterized by coordinated ballistic-like bursts of activity in inspiratory and expiratory muscles. Airflows during intensive coughs can reach 12 L/s in humans [1]. Although it has been proposed that cough and breathing share a common neurogenic control system [2], significant regulatory differences exist between the two behaviors. For example, during eupnea, there are well-known relationships between inspiratory volume (V_I_) and inspiratory time (T_I_) and between expiratory volume (V_E_) and expiratory time (T_E_). Smaller VI or VE are associated with longer TI or TE durations during breathing [3]. This volume timing behavior is mediated by slowly adapting pulmonary stretch receptors (PSR) However, Romaniuk et al [4] suggested that phasic PSR afferent feedback does not play an important role in the development of cough. This suggestion was supported by our previous study in which we found that there was no relationship between volume and phase durations during repetitive tracheobronchial coughing in spontaneously breathing cats [5]. These observations indicate that the regulation of cough motor pattern is fundamentally different than that of breathing. It follows that presumptions of how the cough motor pattern is controlled that are based on our knowledge of the control of the pattern of breathing may be subject to significant error.

In preliminary experiments, we observed that a period of expiratory motor quiescence existed between the end of the expiratory motor burst and the onset of the next inspiration during repetitive cough, consistent with the existence of two subphases within the cough expiratory period [4,6], as first proposed by Romaniuk et al [4]. The presence of two subphases within the expiratory interval of cough is consistent with the control of the expiratory interval during breathing, and if substantiated, would be consistent with the synaptic network model of Shannon and coworkers for cough [2] which accounts for expiratory motor discharge that occurs largely restricted in the early portion of the expiratory phase. However, the extent to which this network model can fully account for spatiotemporal features of the cough motor pattern is not well understood. A significant limiting factor in testing this model is the relative lack of experimental information regarding the control of cough phase durations and intensity. In this study, we investigated the spatiotemporal features of the cough motor pattern during repetitive coughs. We hypothesized that the expiratory period during cough is composed of two subphases each of which is regulated separately. Furthermore, we speculated that the duration of the expiratory interval is a primary determinant of the total cough cycle time.

## Methods

Fifteen cats (3.6 ± 0.3 kg) were anesthetized with pentobarbital sodium (35 mg/kg iv). Supplemental anesthetic was administered when necessary (5 mg/kg, iv). Atropine sulfate (0.1 mg/kg, iv) was administered to block reflex airway secretions. The trachea, femoral artery, and femoral vein were cannulated in all animals. The animals were allowed to spontaneously breathe room air. Blood pressure (mean 139 ± 5 mm Hg) and body temperature were continuously monitored. End-tidal PCO_2 _was continuously monitored all animals but only recorded (36 ± 1 mm Hg) in 11/15 animals. Body temperature was controlled by a heating pad and maintained at 37.5 ± 0.5°C.

Electromyograms (EMG) of respiratory muscles were recorded with bipolar insulated fine wire electrodes by the technique of Basmajian and Stecko [7]. EMGs were recorded from the transversus abdominis or rectus abdominis (ABD, expiratory) muscles and parasternal (PS, inspiratory) muscles. The PS electrodes were placed at T3 proximal to the sternum after exposing the ventral surface of the muscle. Transversus abdominis electrodes were placed 3-4 cm lateral to the linea alba. Rectus abdominis electrodes were placed about 1 cm lateral to the linea alba. Proper placement of each set of electrodes was confirmed by the appropriate inspiratory or expiratory phased activity during breathing and/or cough.

Repetitive tracheobronchial (TB) coughs were elicited by mechanical stimulation of the intrathoracic trachea with a thin flexible polyethylene cannula [8,9]. For TB stimulation, the cannula was introduced into the extrathoracic trachea and advanced so that its tip was at the approximate location of the carina. The cannula was rotated at 1-2 Hz and retracted and advanced repeatedly across a distance of approximately 2 inches during the stimulus trial. However, movement of the trachea during coughing may have resulted in significant variations in how the cannula contacted the airway mucosa during the stimulus trials. Each cough stimulus lasted for 10 seconds. One to three minutes elapsed between stimulus trials.

Cough was defined as a sequence of a large burst in PS muscle EMG followed by a burst in ABD muscle EMG [8]. These criteria distinguished cough from other airway defensive behaviors such as expiration reflex [10,11], augmented breath [12], and aspiration reflex [13,14].

**Figure 1:**
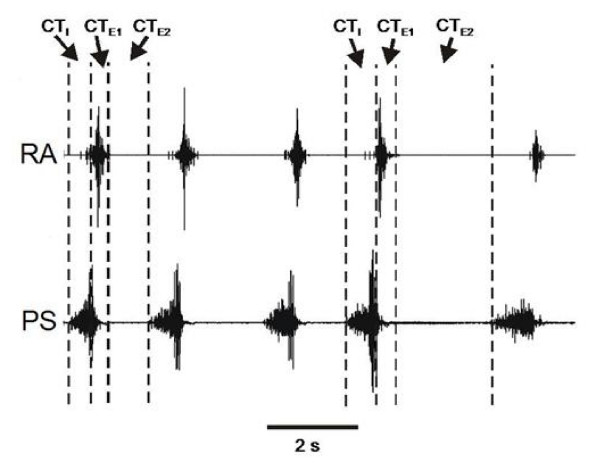
**An example of individual phase duration relationships during a repetitive series of TB coughs**. CT_I _- cough T_I _, CT_E _- cough T_E _(the sum of CT_E1 _and CT_E2_), CT_TOT _- total cough cycle time (the sum of CT_I _CT_E1 _and CT_E2_), CT_E1 _- cough expiratory subphase E1, CT_E2 _- cough expiratory subphase E_2_. Note CT_E2 _and CT_TOT _vary by over 100% in the selected cough cycles while CT_I _and CT_E1 _change very little. RA EMG- rectus abdominis (expiratory) muscle electromyogram, PS EMG-parasternal (inspiratory) muscle electromyogram.

All EMGs were amplified, rectified, filtered (300-5000 Hz), and integrated (time constant 100 ms). The amplitude of the ABD muscle EMG, amplitude of the PS muscle EMG, cough inspiratory (CT_I_) and expiratory (CT_E_) durations were obtained from the moving averages of the EMGs. The PS and ABD muscle amplitudes were normalized to their peak amplitudes during cough in each animal. The phases of cough are illustrated in Figure 1. CT_I _is the duration from the onset to the peak of PS EMG burst. CT_E _was defined as the duration from the peak of PS EMG burst to the onset of the next PS EMG burst. CT_E _was further subdivided into two subphases CT_E1 _defined as the period of the expiratory muscle motor burst during cough and CT_E2_, a period of motor quiescence flowing the expiratory muscle motor burst. CT_TOT _is the duration from the onset of one PS EMG burst to the onset of the next PS EMG burst.

Results are expressed as mean values ± SD. Data were analyzed by linear regression to determine the relationships between cough phase durations and amplitudes. The runs test was used to evaluate linearity of the data. We suggested based on our findings in the cat [15] that the anterolateral abdominal muscles acted as a unit during cough. As such, the normalized data from both abdominal muscles were pooled for the correlation analysis. Multiple regression analysis (stepwise regression) was performed to identify primary determinants of the cough cycle time, in which CT_TOT _was applied as the dependent variable and CT_I_, CT_E1_, CT_E2_, inspiratory EMG amplitude, and expiratory EMG amplitude were the independent variables. For clarity, the squares of linear regression correlation coefficients were designated as r^2^, and multiple regression coefficients of determination were designated as R^2^. Multiple collinearity analysis identified these variables as unrelated to one another. CT_E _was not included in the multiple regression model because multiple collinearity analysis identified this variable as strongly related to CT_E2_. To identify the relative contributions of each independent variable to the variance in CT_TOT_, we conducted a stepwise exclusion protocol in which each of these factors were removed from the dataset and the R^2 ^recalculated [16]. Thus, the contribution of each variable to the variability in CT_TOT _could be inferred.

## Results

A total of 1093 tracheobronchial coughs were elicited in 15 animals. Repetitive tracheobronchial coughing was characterized by sequential inspiratory and expiratory bursting separated during the expiratory phase of each cough cycle by intervals of relative motor quiescence (Fig. 1). These motor quiescent intervals had highly variant durations, even during an ongoing series of repetitive coughing (Fig. 1). Based on these observations, we have separated the cough cycle into three phases: cough inspiratory (CT_I_), cough expiratory phase 1 (CT_E1_), and cough expiratory phase 2 (CT_E2_). CT_I _is defined by the duration of the inspiratory phase (Fig. 1). CT_E1 _is the period of ballistic-like expiratory motor discharge (Fig. 1) and CT_E2 _is the period of relative motor quiescence between the end of CT_E1 _and the onset of the next CT_I _(Fig. 1). In some cases, tonic activity in ABD EMGs could be observed during CT_E2_, but it was clearly distinguished from the ballistic-like expiratory motor bursting during CT_E1_. Furthermore, tonic activity could sometimes be observed in the ABD EMGs during CT_I_, but this activity was much smaller in amplitude than the ABD burst during CT_E1_. We have observed this expiratory co-activation with inspiratory muscles before and have termed it pre-expulsive activity [15].

For all coughs the mean total cough cycle time was 1.76 ± 0.81 s. Phase durations for cough were: CT_I _= 0.49 ± 0.25 s. CT_E1 _= 0.31 ± 0.16 s, and CT_E2 _± 0.96 ± 0.67 s. The average cough inspiratory amplitude was 49 ± 24%% of maximum. The average ABD EMG amplitude was 51 ± 23% of maximum.

**Figure 2:**
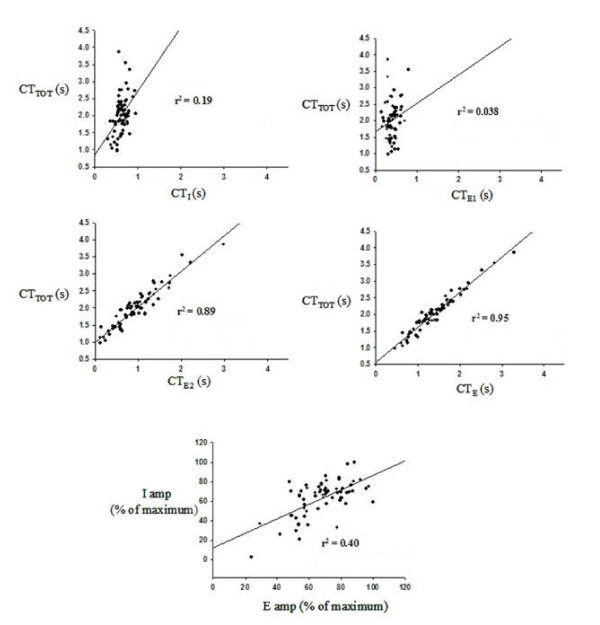
**Regression relationships between cough phase durations and amplitudes during TB coughs from one animal**. Strong linear relationships exist between CT_TOT _and CT_E _and CT_E2 _but CT_I _and CT_E1 _appear to be relatively constant in spite of a 300% variation on CT_TOT_. I amp-inspiratory muscle EMG amplitude, E amp-expiratory muscle EMG amplitude.

Transient increases in the frequency of coughing within a bout were associated with a larger relative decrease in CT_E2 _(Fig. 2). Regression analysis revealed strong linear correlations between CT_TOT _and CT_E2 _(r^2 ^= 0.89 ± 0.04). A weak correlation existed between CT_TOT _and CT_I _(r^2 ^= 0.24 ± 0.05). There were no significant relationships between CT_TOT _and CT_E1 _(r^2 ^= 0.09 ± 0.03), inspiratory (r^2 ^= 0.07 ± 0.02), or expiratory amplitudes (r^2 ^= 0.11 ± 0.03) and CT_TOT _(Table 1). There was only a weak correlation between inspiratory and expiratory amplitudes during cough (r^2 ^= 0.29 ± 0.05, Table 2). Values for r^2 ^for relationships between the other variables were all less than 0.13 (Table 2).

**Table 1:** Correlation coefficients (r^2^) from regression relationships between CT_TOT _and phase durations and EMG amplitudes during repetitive TB coughs in individual animals.

Animal	CT_TOT _Simple Linear Regression Coefficients (r^2^)					
	
	CT_I_	CT_E1_	CT_E2_	CT_E_	I Amp	E Amp
1	0.48	0.01	0.93	0.93	0.02	0.04
2	0.48	0.06	0.87	0.87	0.00	0.04
3	0.20	0.04	0.86	0.86	0.04	0.16
4	0.07	0.46	0.98	0.98	0.02	0.16
5	0.57	0.07	0.90	0.90	0.003	0.05
6	0.24	0.16	0.93	0.95	0.00	0.15
7	0.49	0.02	0.35	0.35	0.05	0.0009
8	0.32	0.0007	0.92	0.96	0.24	0.32
9	0.17	0.10	0.98	0.99	0.19	0.29
10	0.26	0.003	0.88	0.95	0.05	0.02
11	0.05	0.10	0.98	0.99	0.08	0.13
12	0.18	0.17	0.92	0.94	0.04	0.01
13	0.008	0.14	0.94	0.87	0.27	0.30
14	0.001	0.07	0.98	0.97	0.02	0.03
15	0.08	0.017	0.96	0.84	0.06	0.01

	0.24 ± 0.05	0.09 ± 0.03	0.89 ± 0.04	0.89 ± 0.04	0.07 ± 0.02	0.11 ± 0.03

The only high r^2 ^value is for the relationship between CT_TOT _and CT_E2_.

**Table 2:** Average correlation coefficients (r^2^) from regression relationships between cough phase durations and EMG amplitudes during repetitive TB coughs.

	Simple linear regression coefficients for cough phase or EMG magnitude (r^2 ^± SE)				
	
	CT_I_	CT_E1_	CT_E2_	I Amp	E Amp
CT_I_	X	0.03 ± 0.01	0.09 ± 0.02	0.08 ± 0.03	0.05 ± 0.01
CT_E1_	X	X	0.09 ± 0.03	0.04 ± 0.01	0.1 ± 0.03
CT_E2_	X	X	X	0.07 ± 0.02	0.12 ± 0.02
I Amp	X	X	X	X	0.29 ± 0.05

There were only weak correlations between individual phase durations and a moderate relationship between inspiratory and expiratory EMG amplitudes during coughing.

Multiple regression analysis of CT_TOT _to CT_I_, CT_E1_, and CT_E2 _showed that R^2 ^only decreased by 0.08 when CT_I _was excluded from the equation, and 0.034 when CT_E1 _was excluded. This suggested the exclusion of CT_I _had a minimal effect on CT_TOT_. The R^2 ^value decreased by 0.67 when CT_E2 _was excluded from the analysis, suggesting CT_E2 _was the most important contributor to CT_TOT_.

## Discussion

The first major finding of this study was that cough expiratory phase can be divided into two subphases, CT_E1 _and CT_E2_. The second finding of this study was that CT_E_, mainly CT_E2_, is the primary determinant of CT_TOT _. Fluctuations in the duration of CT_TOT _are primarily the result of increases or decreases in CT_E2_. Given that EMG burst amplitudes were not correlated with phase durations during cough, our results also suggest separate regulatory mechanisms for the intensity and cycle durations of cough.

This is the first report to quantify that the expiratory phase during coughing, like that of breathing, can be composed of two phases. This concept was first proposed by Romaniuk and coworkers [4], but some of the temporal relationships that we illustrate in Figure 1 can be seen in figures in studies published by other groups [17,18]. In fact, Korpas and Tomori [18] show figures that suggest that periods of motor quiescence in the expiratory period during repetitive coughing exist in cats (Fig 32, p. 76), rabbits (Fig 42, p. 107), and in a separate study, dogs [19] (Fig 1A). During breathing, the activity patterns of spinal respiratory motoneurons have been used to subdivide the expiratory phase into two phases, the postinspiratory phase (E1) and active expiration phase (E2) [20-25]. The E1 phase of breathing represents the "passive" stage of expiration in which chest wall and abdominal muscles are relatively quiescent. The E2 phase can be associated with "active" expiration in which chest wall and abdominal muscles can exhibit an augmenting discharge [22,26]. Our evidence for the existence of two phases of the expiratory interval during cough is primarily based on observations related to the expulsive motor burst and the existence of a variable duration of the subsequent motor quiescence. The E1 and E2 phases during cough differ significantly from those of breathing. For example, CT_E1 _is demarked by ballistic expiratory motor activation, whereas this phase during breathing represents a period of relative quiescence of expiratory pump muscles [4,26]. During CT_E2_, there is a period of motor quiescence, and during breathing E2 expiratory pump muscles can be very active [4,22].

Our study showed that the duration of the CT_E1 _phase during repetitive coughing is relatively fixed and that the duration of CT_E2 _is variable. Romaniuk reported CT_E _was prolonged during obstructed cough in which the trachea was occluded at the end-inspiration and maintained throughout the subsequent expiration [4]. Our results are consistent with the idea that the enhanced vagal afferent stimulation resulting from airway occlusion has a preferential effect to prolong the duration of CT_E2_.

Poliacek et al. reported [27] that CT_I _during laryngeal coughs was 50% longer than during TB coughs, and the two types of coughing had similar CT_E1 _durations in the present study. In our protocol, bouts of repetitive TB coughs were elicited, whereas Poliacek et al. [27] elicited mostly single coughs. Furthermore, the results of our previous study, showed that CT_I _during single TB coughs or first coughs of a bout is significantly longer than during repetitive coughs [5]. These observations indicate that some features of the motor pattern of coughing can exhibit a high degree of variation depending upon the region of the airway from which it is elicited and whether single or repetitive behaviors are produced. In essence, all coughs are not the same, even within a series of repetitive coughing. However, selected components of the cough motor pattern are fixed, such as the duration of CT_E1_.

The lack of relationship between inspiratory and expiratory motor burst amplitudes differs from that reported previously for the fictive cough model in the cat by our group [28]. In that study, we showed that there was a linear relationship between inspiratory and expiratory neurogram amplitudes during fictive cough that was disrupted by codeine. The effect of codeine was manifest at doses that did not significantly suppress either inspiratory or expiratory amplitudes, but were sufficient to reduce cough number. The results of that study were consistent with the existence of a neurogenic mechanism for coordinating inspiratory and expiratory motor drive during coughing that was separate from simple inhibition of excitatory motor drive to one or both of the motoneuron pools. In the fictive model, cough is produced in the absence of active or passive muscle movement in decerebrated, paralyzed animals [2,9,13]. Therefore, the contribution of sensory feedback from active muscle movement to the cough motor pattern generator is eliminated. The rate of lung inflation during cough in the fictive cough model is typically similar to that during fictive breathing and peak lung volume is likely to be less than that produced in spontaneously breathing animals, presumably resulting in altered pulmonary afferent feedback. It is conceivable that these differences in somatic and pulmonary afferent feedback this may cause changes in the cough motor pattern in the fictive model relative to the spontaneously breathing preparation. However, we believe that the absence of a coordinating mechanism between inspiratory and expiratory motor drive in spontaneously breathing animals is most likely related to the presence of anesthesia. Sodium pentobarbital was used in the present experiments and this anesthetic has been successfully employed in cough studies for many years [13,18,29]. Cats are capable of producing intense coughing while anesthetized with sodium pentobarbital.

Our results are consistent with the concept that the synaptic model of Shannon and coworkers can account for expiratory phase durations during cough. In Shannon's model, the expiratory augmenting (E-Aug) neurons in the Botzinger complex consist of at least two subpopulations based on their discharge patterns during cough [2]. As such, these synaptic relationships governing the discharge patterns of rostral ventral respiratory column expiratory neurons could account for a cough expiratory interval composed of two subphases. Our results are significant in that they show that the expiratory interval during cough is, in fact, controlled in this fashion. Furthermore, our findings extend our understanding of the regulation of the motor pattern of respiratory muscles by the respiratory pattern generator.

It is not clear how the model of Shannon and coworkers can account for a fixed CT_E1_, while CT_E2 _is highly variant. Our data showed that the CT_E1 _was independent of ABD burst intensity, CT_TOT_, CT_E_, and the previous CT_I_. Our data also indicate that the duration of CT_E2 _determines CT_TOT _length. Based on these observations and inspection of the model of Shannon and coworkers, when the frequency of repetitive cough is increased (i.e. CT_E2 _and thus CT_TOT _decreased), inspiratory decrementing neurons should have a stronger inhibition on the activity of the E-Aug late neurons, an action which would shorten CT_E2_. But the model cannot answer the question why CT_E1 _duration is not also reduced when CT_E2 _decreases by 50% or more (Fig 1). Our observation that CT_E1 _is relatively invariant indicates that this phase also has an upper limit in duration.

Correlation analysis showed that there was no relationship between cough expiratory amplitude and CT_E1 _duration. Similarly, there was no correlation between the inspiratory amplitude and CT_I_. These results are consistent with a previous study, showing there was no relationship between expiratory volume and CT_E_, or between inspiratory volume and CT_I _[5]. These observations are not consistent with what is predicted from Shannon's model. According to this model, input from rapidly adapting receptor relay neurons excites neurons that regulate both inspiratory and expiratory phase durations as well as E-Aug early neurons, expiratory premotor neurons, and inspiratory augmenting premotor neurons that that provide excitatory motor drive to spinal expiratory and inspiratory motor pathways. This feature of the model suggests that the magnitude of expiratory motor activation during cough should be related to expiratory phase duration, and the magnitude of inspiratory motor activation should be related to inspiratory phase duration.

It should be noted that the cats in our preparation were spontaneously breathing whereas Shannon's experiments were based on a fictive cough model. In the fictive model, cough was produced in the absence of active or passive muscle movement in decerebrated, paralyzed animals [28,30,31]. Therefore, the contribution of sensory feedback from active muscle movement to the cough motor pattern generator was eliminated. The rate of lung inflation during cough in the fictive cough model is typically similar to that during fictive breathing and peak lung volume is likely to be less than that produced in spontaneously breathing animals, presumably resulting in altered pulmonary afferent feedback. It is conceivable that these differences in somatic and pulmonary afferent feedback may cause changes in the cough motor pattern in the fictive model relative to the spontaneously breathing preaparation. Furthermore, we stimulated repetitive cough whereas Shannon used single cough stimulation. It has been reported that the first cough in a series or a single cough compared to repetitive coughs has different cough motor patterns [5].

## Conclusions

Our findings provide information regarding the functional organization of the central neurogenic mechanism for cough. Reconfiguration of the respiratory pattern generator to produce coughing not only changes the arrangement of the respiratory neural network but it also changes fundamental features that govern how the motor pattern is controlled. Cough and breathing differ in that: a) motor drive and phase durations are controlled separately for cough, and b) the E2 subphase is the dominant regulator of total cycle duration for cough.

## Abbreviations

ABD: abdominal; CT_I_: cough inspiratory time; CT_E_: cough expiratory time; CT_E1_: first segment of cough expiratory phase; CT_E2_: second segment of cough expiratory phase; CT_TOT_: total cough cycle time; E1: postinspiratory phase of breathing; E2: active expiratory phase of breathing; E-Aug: expiratory augmenting neuron; EMG: electromyogram; E-amp: expiratory amplitude; I-amp: inspiratory amplitude; PC02: partial pressure of exhaled carbon dioxide; PSR: pulmonary stretch receptor; PS: parasternal muscle; RA: rectus abdominis; SD: standard deviation; TB: tracheobronchial; T_E_: breathing expiratory time; T_I_: breathing inspiratory time; V_E_: expired volume during breathing; V_I_: inspired volume during breathing.

## Competing interests

The authors declare that they have no competing interests.

## Authors' contributions

CY performed experiments, conducted data analysis and interpretation, and participated in writing the manuscript. SS conducted statistical analysis of the data. MJR performed experiments and conducted data analysis. PWD interpreted the data and edited the manuscript. DCB performed experiments, interpreted the data, and participated in writing the manuscript. All authors have read and approved the final manuscript.
